# Mortality risk factors in patients with gastric cancer using Bayesian and ordinary Lasso logistic models: a study in the Southeast of Iran 

**Published:** 2020

**Authors:** Abolfazl Hosseinnataj, Mohammad RezaBaneshi, Abbas Bahrampour

**Affiliations:** *Modeling in Health Research Center, Faculty of Health, Institute for Futures Studies in Health, Kerman University of Medical Sciences, Kerman, Iran*

**Keywords:** Gastric cancer, Risk factors, Iran, Lasso regression, Bayesian

## Abstract

**Aim:**

The aim of this study was to apply two types of statistical models to determine the factors that influence the mortality rate in patients with gastric cancer.

**Background:**

In Iran, gastric cancer ranks the first and second most prevalent among men and women, respectively. It is the first cause of death in Iran in both gendersival.

**Methods:**

In this retrospective study, data were obtained from 339 (216 male) patients diagnosed with gastric cancer in the city of Kerman (South-East of Iran) during 2001-2015. In this study, ordinary and Bayesian Lasso (least absolute shrinkage and selection operator) logistic regression models, with goodness-of-fit indices, were compared and the models’ risk factors were also determined.

**Results:**

The mean age of the participants was 62.84 ±14.53 years, and 12.4% of them were younger than 45 years. Also, the mortality rate was 57.7%. Gender, morphology of the tumor, and time of diagnosis were found to be significant factors in the mortality of the patients in both models. This study found that the Bayesian Lasso model had better fitness.

**Conclusion:**

The high mortality rate of gastric cancer and its high prevalence at age below 45 years are alarming. Thus, great attention should be paid to prevention, early diagnosis, especially in females, and adenocarcinoma to improve the survival of patients with gastric cancer.

## Introduction

 Cancer is one of the main causes of death worldwide; the total number of its deaths was estimated to be around 9.6 million in 2018 (1). Among all types of cancer, gastric cancer is the fourth common cancer in men and the fifth in women, with nearly 50% of new cases of gastric cancer occurring in developing countries (2-4).

In Iran, gastric cancer ranks the first and second among men and women, respectively (5). The incidence of this cancer has beren reported to be about 7300 cases per year in Iran (6). It is the most fatal cancer in Iran in both sexes (5). In a study by Khoshdel and et al, gastric cancer data from 2007 to 2017 were used to predict new cases until 2019. The results of this study revealed that the incidence of gastric cancer in Iran has been increasing overall, but its mortality has been constant (7). The one- and five-year survival rates of gastric cancer are estimated 52% and 15% respectively (8). In Kerman (South-East of Iran), the one- and five-year survival rates are estimated 62% and 45% respectively (9).

Gastric cancer does not usually have symptoms in the first years; thus, the diagnosis of this disease does not usually occur in the early stages of the disease, whereby the odds of death increases (10). Hence, early detection of the disease can play an important role in reducing mortality. On the other hand, considering the annual increase in the number of cases of gastric cancer, higher mortality rate than other cancers, as well as the changes in risk factors with respect to the time and place of the population, it is of paramount importance to detect the risk factors that increase the mortality rate of patients with gastric cancer.

Two main challenges in the modeling are enough sample size and multi-collinearity among exploratory variables. One of the popular methods that can address these issues is Lasso regression model. In the area of cancer for example, there are many data sets that have a large number of risk factors given the advances in technology and accurate instruments; these data sets have usually a small sample size and multi-collinearity challenges.

In this study, we applied the lasso regression model due to multi-collinearity between some explanatory variables (the maximum value of correlation was equal to 0.8). On the other hand, an important feature of Bayesian method is controlling the uncertainty of the parameters by choosing a prior distribution for them. Having reviewed the previous studies, it became clear that the Bayesian Lasso logistics model has been used less frequently, and in this study it was used for the first time. The objectives of this study were to determine the factors that influence the mortality of gastric cancer patients and to compare Bayesian and non-Bayesian lasso models to choose the best one. 

## Methods

**Patients**

In this analytical retrospective study, the data were obtained from all patients who were diagnosed with cancer and had been admitted in 2 hospitals (Bahonar and Afzalipoor hospitals) in Kerman during 2001-2015. The data were collected by checking the hospital documents in 2017. Some of the information that was not available in the documents was collected through phone calls. To verify the accuracy of the data, they were adapted to Information of the Cancer Registry Center. After removing duplications, the extracted data included 339 patients with positive gastric cancer. The patients' death status was recorded at the end of the study.

Patient’s death status (alive=0, dead=1) was considered as a dependent variable. Covariates included age (year), gender (male or female), smoking (no or yes), addiction to opium (no or yes), place of residence (rural or urban), surgical history (no or yes), chemotherapy history (no or yes), radiotherapy history (no or yes), histological grade (well, moderate or poor), cancer staging (1, 2, 3 or 4), tumor morphology (neoplasm, carcinoma, and adenocarcinoma), metastasis (no or yes), family history (no or yes), and date of diagnosis to death or end of research (month).

**Statistical Analysis**

The conditions for using Lasso regression are small sample size and co-linearity between variables. The sum of the least squares error in the Lasso for linear regression is as follows:

${\mathrm{RSS}}_{L}=\sum_{i=1}^{n}{\left(y_{i}-\beta_{0}-\beta_{1}x_{1}-\ldots -\beta_{p}x_{p}\right)}^{2}+\lambda \sum_{j=1}^{p}\left|\beta_{j}\right|=\mathrm{RSS}+\lambda \sum_{j=1}^{p}\left|\beta_{j}\right|$

In this formula, y is the response variable, x_j_ represents the vector of the independent variable values. In the penalty term $\lambda \sum_{j=1}^{p}\left|\beta_{j}\right|$ which called L1-norm, β_j_ shows the regression coefficient for j^th^ variable and λ represents tuning parameter.

Regression coefficients are obtained by minimizing the above formula. The tuning parameter plays an important role in the results of Lasso regression. By increasing λ, more coefficients of the model will be zero; λ = 0 is similar to ordinary regression and all coefficients remain in the model. Typically, the tuning parameter is calculated through cross-validation (11, 12).

In this study, two models were used to analyze gastric cancer data: (1) Lasso logistic regression model, with a constant value of the tuning parameter calculated through the 10-fold cross validation and (2) Bayesian Lasso logistic regression model by specifying a prior distribution for the tuning parameter.

The Bayesian Lasso model:

y~bin(n,p)

$p=\frac{\exp\left(\mathrm{X\beta}\right)}{1+\exp\left(\mathrm{X\beta}\right)}$

β|tau~N(0,τ)

$\tau |\lambda \sim \prod \frac{\lambda^{2}}{2}\exp\left(-\frac{\lambda^{2}\tau^{2}}{2}\right)d\tau^{2}$

$\lambda^{2}\sim \prod \frac{\delta^{r}}{\Gamma (r)}{\left(\lambda^{2}\right)}^{r-1}\exp(-\delta \lambda^{2})d\lambda^{2}$

Where, r=δ=10. This model generalizes the model Park and Casella proposed for quantitative outcome (13). To estimate the parameters in this model, initial values are considered first for each parameter. For the next iteration, parameters will be simulated hierarchically. This algorithm will continue until the parameters converge. In ordinary Lasso, the regression coefficients are equal to zero for non-significant variables. This advantage does not exist in the Bayesian approach, and usually a credible interval is used to determine significant variables (14).

**Comparison of models**

Indicators including Extended Bayesian Information Criteria (EBIC), Akaike Information Criterion (AIC), sensitivity, and specificity were applied to compare the performance of models. The data were divided into 2 parts: train (60%) and test (40%). Models were fitted on the train set while the indicators were calculated on the test set.

The parameters were produced by Gibbs sampling in Bayesian approach. Also, 95% credible intervals were used to select significant variables. For this purpose, a regression coefficient whose credible interval included zero was considered zero. On the other hnad, for invariables whose credible interval did not include zero, the median was used as the estimate of coefficients.

To ensure sampling reliability, all the processes mentioned above were repeated 100 times with the average of the indicators used to compare the models. The analyses were performed using the R software (glmnet packages and programming).

**Simulation study**

We investigated the performance indices of two models across two simulation scenarios. For both scenarios, we simulated a data set with n= (50,100,150) for the train dataset to fit models, and half of them for the test dataset to compare the performance of proposed models. The pairwise correlation between x_i_ and x_j_ was set to be corr(x_i_,x_j_)=ρ^|i-j|^, where ρ=(0.2,0.8). Indicators EBIC, AIC, accuracy ((true positive+ true negative) /total) and precision (true positive/ (true positive+ false positive)) were applied to compare models goodness of fit. Other methods of model implementation were the same as the instructions implemented in gastric cancer data.

We considered two scenarios for constructing the datasets in which binary outcome variable was built based on given coefficients. In the first scenario, the dataset consist of eight independent variables with coefficients β=(3,1.5,0,0,2,0,0,0). In the second scenario, we applied nine independent variables (4 binary and 5 continues variables) with coefficients β=(2,-1,0,0,1,-2,0,0,0). 

## Results

In this study, 339 patients were enrolled, of whom 216 were male (63.7%). The mean age of the participants was 62.84 ±14.53 years and the median age was 64 years. Approximately, 12.4% of the patients were younger than 45 years and 11% were older than 80 years. Mortality rates in individuals under 45 and over 45 years were 52.4% and 58.2%, respectively.

Of all participants, 195 (57.5%) died before the end of the study. Table 1 reports the descriptive information of some variables related to death status. 

On average, 3 and 1.5 variables were significant at 100 repeats in the ordinary and Bayesian models, respectively. Thus, in the ordinary Lasso, three variables with the greatest frequent nonzero estimated coefficients were considered as factors affecting the death of patients with gastric cancer. Also, in the Bayesian Lasso model, two variables were identified that had the maximum number of nonzero coefficients. 

In the ordinary Lasso model, sex, tumor morphology, and duration of diagnosis and in the Bayesian Lasso model, tumor morphology and duration of diagnosis had the most frequent estimated nonzero coefficients compared to other factors. Table 2 reports the beta, odds ratio (OR), and 95% interval of each of the variables for the two models.

In the Lasso logistic regression, the odds of death for females were 2.64 times that of males. The patients with adenocarcinoma tumor morphology were 86% less likely to die than with neoplasm (OR=.14). Also, the odds of death declined as the duration of diagnosis increased (OR=0.21). 

In the Bayesian Lasso logistic regression, patients with adenocarcinomas were less likely to die than those with neoplasms (OR=0.25). Further, the odds of death diminished as the duration of diagnosis increased (OR=0.3).

**Table 1:** Frequency of some variables related to death status

Variable		Total (%)	Alive (%)	Died (%)
Sex	Men	216 (63.7)	95 (66)	121 (62)
	Women	123 (36.3)	49 (34)	74 (38)
Place of residence	Rural	67 (19.8)	33 (22.9)	34 (17.4)
	Urban	272 (80.2)	111 (77.1)	161 (82.6)
Smoking status	Yes	91 (26.8)	38 (26.4)	53 (27.2)
	No	248 (73.2)	106 (73.6)	142 (72.8)
Grade	Well	11 (3.2)	5 (3.5)	6 (3.1)
	Moderate	270 (79.6)	119 (82.6)	151 (77.4)
	Poor	58 (17.1)	20 (13.9)	38 (19.5)
Stage	I	9 (2.7)	3 (2.1)	6 (3.1)
	II	205 (60.5)	94 (65.3)	111 (56.9)
	III	91 (26.8)	35 (24.3)	56 (28.7)
	IV	34 (10)	12 (8.3)	22 (11.3)
Tumor morphology	Neoplasm	23 (6.8)	3 (2.1)	20 (10.3)
	Carcinoma	53 (15.6)	21 (14.6)	32 (16.4)
	Adenocarcinoma	263 (77.6)	120 (83.3)	143 (73.3)

Percentages calculated by column

**Table 2:** The estimated coefficients in the two models

Model	Variable		beta	OR	95% CI OR
Lasso logistic	Sex	Female	0.97	2.64	(1.22-5.87)
		Men		ref	
	Tumor morphology^*^	Adenocarcinoma	-1.96	0.14	(0.02-0.62)
		Neoplasm		ref	
	Duration of diagnosis	-	-1.55	0.21	(0.13-0.33)
Bayesian Lasso logistic	Tumor morphology^*^	Adenocarcinoma	-1.4	0.25	(0.08-0.85)
		Neoplasm		ref	
	Duration of diagnosis	-	-1.19	0.3	(0.15-0.37)

* The carcinoma was not sig

nificant.

**Table 3:** Comparison of the two models

Model	EBIC	AIC	Sens	Spec	Mean(λ)
lasso	205.1	196	.7	.62	.04
Bayesian Lasso	191.8	187.6	.79	.60	.67

; AIC=-2logL+2p; Sens=TP/(TP+FN);

The indicators for comparing the two models are presented in Table 3, demonstrating that the indicators for the Bayesian Lasso were better. The mean of λ in ordinary Lasso and Bayesian Lasso was .04 and .67, respectively, based on cross-validation. Also, the value of λ was greater in the Bayesian model.

**The Simulation results**

In the first scenario, the Bayesian model had the best performance. As the sample size increased, the performance of models became almost the same, but the performance of models diminished as the correlation between variables increased. The first and second scenarios produced the same results. In the second scenario, two models showed almost the same performances, but Bayesian model had the best precision index.

## Discussion

Gastric cancer commonly affects people over 45 years. In this study, the proportion of patients under 45 years with gastric cancer was almost the same as in another study conducted in Iran (11.8%) (15) and more than in the United States (8.3%) (16). The present study revealed a mortality rate of 57.5% among patients with gastric cancer, which is far larger than other studies (7, 17) and was the same as in Hormozgan (18). The possible reasons for the high mortality rate in our study are probably transfer of end stage cancer patients from neighboring provinces (such as Sistan and Baluchistan) or the longer follow-up time in our study than in other studies.

Overall, in this study, factors including sex, tumor morphology, and duration of diagnosis influenced the death of gastric cancer patients in the two models.

Although 62.1% of the patients were men, women had a higher odds of death. Women were more likely to die from gastric cancer in comparison to men. In a study in Iran, a similar result was found, in which the proportion of men (76%) was higher than that of women, while the likelihood of death was higher in women (19, 20). However, in another study, the likelihood of death was higher among male patients (21, 22). One possible explanation for this result might be the occupational exposure which may contribute to increased gastric cancer incidence in males (23). On the other hand, higher fatalities among women may be associated with the nutrition, obesity, or genetics (24).

The odds of death in patients with adenocarcinoma was 86% less than that of those with neoplasm. Similar to other studies, in this study, the highest frequency was related to adenocarcinoma (15, 19, 25), with tumor morphology identified as an effective variable in the death of patients with gastric cancer (26, 27). Also, in another study, conducted with the same data but via other statistical methods, the morphology was found to be one of the important factors influencing the survival of the patients with gastric cancer (28).

The duration of diagnosis was also a significant factor in the mortality of the patients. The results of this study showed that the odds of death diminished as the duration of diagnosis increased. This result may be due to detecting the disease in the early stages in some patients, and thus reducing the chance of death by applying appropriate and timely treatments (29). Also, another reason is the fatality of this disease in its early stages, and thus if patients can survive this period, their chance of survival increases (30).

By comparing the ordinary and Bayesian methods of Lasso logistic regression, it was found that the Bayesian method had a better performance. Bayesian method can better control the uncertainty of the parameters by determining the prior statistical distribution. In a study by Korhani et al. on the same data, ordinary and Bayesian neural networks were compared and they found that the Bayesian method had a better performance. Also, they observed that age, histological grade, and morphology were the most important factors in predicting the death of patients. In their study, a higher sensitivity and specificity was obtained for Lasso models compared to the present study (28). The lasso model reduces the coefficients of some variables towards zero and sets the coefficients of insignificant variables equal to zero. Because of the mentioned properties, the standard deviation of regression coefficients cannot be calculated by the usual methods, but the standard deviation and credible interval can be calculated in the Bayesian methods based on Markov chain Monte Carlo (MCMC) , which is one of the benefits of this model. In contrast to ordinary lasso models, insignificant coefficients are not exactly equal to zero in the Bayesian methods, so automatic variable selection property is not established. However, one of the main problems of Bayesian method is the complexity of its calculation, and thus researchers use it less often.

The high mortality rate of gastric cancer and its high prevalence at ages younger than 45 years are alarming. Thus, great attention should be paid to prevention and early diagnosis through screening, especially in females, as well as tumor morphology to decrease the chance of death among this group of patients. 

Further, the use of the Bayesian approach can help improve the model fitness; thus, it is highly recommended that the proposed model be evaluated for medical research.
